# Diagnostic Accuracy of Non-Radiologist-Performed Ultrasound for Diagnosing Acute Appendicitis in Pediatric Patients: A Systematic Review and Meta-Analysis

**DOI:** 10.3390/medicina61071308

**Published:** 2025-07-21

**Authors:** Se Kwang Oh

**Affiliations:** 1 Department of Emergency Medicine, Chungnam National University Sejong Hospital, Sejong 30099, Republic of Korea; 13744@hanmail.net or skoho@cnuh.co.kr; Tel.: +82-44-995-3021; 2 Department of Emergency Medicine, College of Medicine, Chungnam National University, Daejeon 34131, Republic of Korea

**Keywords:** appendicitis, child, ultrasonography, systematic review

## Abstract

*Background and Objectives:* Acute appendicitis is a common cause of abdominal pain requiring surgery in pediatric patients. Given concerns regarding radiation exposure from computed tomography (CT), ultrasound (US) has become the first-line diagnostic modality. In many emergency and resource-limited settings, non-radiologist physicians often perform these examinations. This study aimed to evaluate the diagnostic accuracy of a non-radiologist-performed ultrasound in detecting acute appendicitis in children. *Materials and Methods:* We conducted a systematic review and meta-analysis according to the PRISMA guidelines. The literature was searched across PubMed, Ovid MEDLINE, EMBASE, the Cochrane Library, and Google Scholar through June 2024. Studies reporting on the sensitivity, specificity, positive predictive value (PPV), and negative predictive value (NPV) of non-radiologist-performed ultrasounds in pediatric appendicitis were included. Study quality was assessed using the QUADAS-2 tool, and a bivariate random-effects model was used for statistical analysis. *Results:* Eight studies, with a total of 1006 pediatric patients, were included. The pooled sensitivity and specificity were 0.87 (95% CI, 0.83–0.90) and 0.93 (95% CI, 0.91–0.95), respectively. The area under the SROC curve was 0.783 (95% CI, 0.708–0.853), suggesting moderate-to-good diagnostic accuracy. Substantial heterogeneity was observed across studies, possibly due to differences in operator training and ultrasound techniques. *Conclusions:* Non-radiologist-performed ultrasound demonstrates moderate-to-good diagnostic accuracy in identifying pediatric appendicitis. These findings support its implementation in emergency or resource-constrained settings and suggest that incorporating structured ultrasound training for non-radiologists may improve timely diagnosis and optimize clinical decision-making in pediatric emergency care.

## 1. Introduction

Each year, nearly 70,000 children in the U.S. are diagnosed with appendicitis, making it a common surgical emergency in pediatric abdominal pain cases [1,2]. The diagnosis of acute appendicitis can be difficult due to its atypical presentations, particularly in pediatric patients [3]. Due to concerns about radiation exposure associated with computed tomography (CT), ultrasound (US) has become the preferred primary diagnostic tool for evaluating appendicitis in pediatric patients. Accordingly, the American College of Emergency Physicians and the American College of Radiology recommend the use of ultrasound as a first-line imaging modality for diagnosing appendicitis in children [4,5,6]. The limited availability of radiology specialists, particularly during weekends and nighttime hours, often complicates the provision of timely diagnostic evaluations in emergency settings. Consequently, non-radiologists, including emergency physicians, commonly perform ultrasound examinations to diagnose acute appendicitis. According to several studies, the accuracy of ultrasound examinations performed by emergency medicine physicians and radiologists in diagnosing acute appendicitis has been reported to be similar [7,8,9]. There have also been several studies on the accuracy of non-radiologist performed ultrasound for the diagnosis of acute appendicitis in pediatric patients [8,10,11,12,13,14,15,16].

Recently, diagnostic scoring systems that integrate ultrasonographic findings with clinical and laboratory parameters have been introduced. Notably, Arredondo Montero et al. proposed the BIDIAP index, which combines sonographic, clinical, and analytical criteria to improve diagnostic accuracy in children with suspected acute appendicitis [17].

In addition, several novel ultrasonographic parameters have been identified as potentially valuable in the diagnosis of appendicitis. For example, the use of the sum of the cross-sectional diameters of the appendix has been reported to enhance diagnostic accuracy compared to single-diameter measurements [18]. Furthermore, the application of color Doppler imaging to assess appendiceal blood flow has shown potential to improve specificity, particularly in cases involving borderline-sized appendices [19]. Another promising addition is the Appendicitis Inflammatory Response (AIR) score. In a recent study, it was validated in a cohort of 184 children, showing that AIR ≥ 9 effectively predicts perforated appendicitis (AUC 0.80, sensitivity 89.5%, specificity 71.9%), providing a complementary risk stratification tool alongside ultrasound [20].

These recent developments reflect the evolving role of ultrasonography in appendicitis diagnosis and underscore the need to evaluate its diagnostic performance when performed by non-radiologist clinicians.

Despite guideline recommendations favoring the use of ultrasound, there remains a notable gap between ideal imaging protocols and real-world clinical practice, especially in emergency departments with limited resources or high patient volumes. Furthermore, the diagnostic accuracy of ultrasound for pediatric appendicitis is highly operator-dependent and may be affected by various factors, such as patient body habitus and the presence of bowel gas. In addition, ultrasound examination in pediatric patients presents unique challenges compared to adults, primarily due to smaller body habitus and limited patient cooperation. These factors necessitate not only greater familiarity with pediatric-specific anatomical variations but also a higher degree of operator skill and experience in performing sonographic evaluations.

While the diagnostic utility of ultrasound for acute appendicitis has been well-studied in adults, the body of evidence focusing on pediatric populations remains relatively limited. Moreover, studies investigating the diagnostic performance of ultrasound when conducted by non-radiologist practitioners in children are particularly uncommon.

Thus, this systematic review and meta-analysis aims to assess how accurately non-radiologists can use ultrasound to diagnose acute appendicitis in children.

## 2. Materials and Methods

### 2.1. Study Design

We systematically reviewed studies on the diagnostic accuracy of non-radiologist performed ultrasound for the diagnosis of acute appendicitis in pediatric patients. This systematic review was conducted according to the Preferred Reporting Items for Systematic Reviews and Meta-Analyses (PRISMA) guidelines [21]. This systematic review was not registered in PROSPERO or any similar publicly accessible database. However, the review was conducted in accordance with the PRISMA guidelines.

### 2.2. Data Sources and Search Strategy

A systematic literature search was conducted in PubMed, Ovid MEDLINE, EMBASE, the Cochrane Library, and Google Scholar up to June 2024. The search strategy included a combination of MeSH terms and free-text keywords, as follows: (“ultrasound” OR “ultrasonography” OR “sonography” OR “US” OR “USG”) AND (“appendicitis” OR “appendix”) AND (“child” OR “children” OR “pediatric”). Boolean operators (AND, OR) were applied appropriately. No restrictions were placed on keyword combinations or publication dates in the title or abstract fields. The search was restricted to articles published in English. Study selection and data extraction were performed by the sole author.

### 2.3. Inclusion Criteria and Selection of Studies

We reviewed the titles and abstracts of the extracted articles, and then examined the full texts of those potentially relevant to the research topic. Studies evaluating non-radiologist-performed ultrasound for diagnosing acute appendicitis in pediatric patients were included. If the ultrasound results were positive, surgical pathology was used as the diagnostic reference standard; if negative, clinical follow-up was used.

### 2.4. Literature Search Outcomes

**Figure 1:**
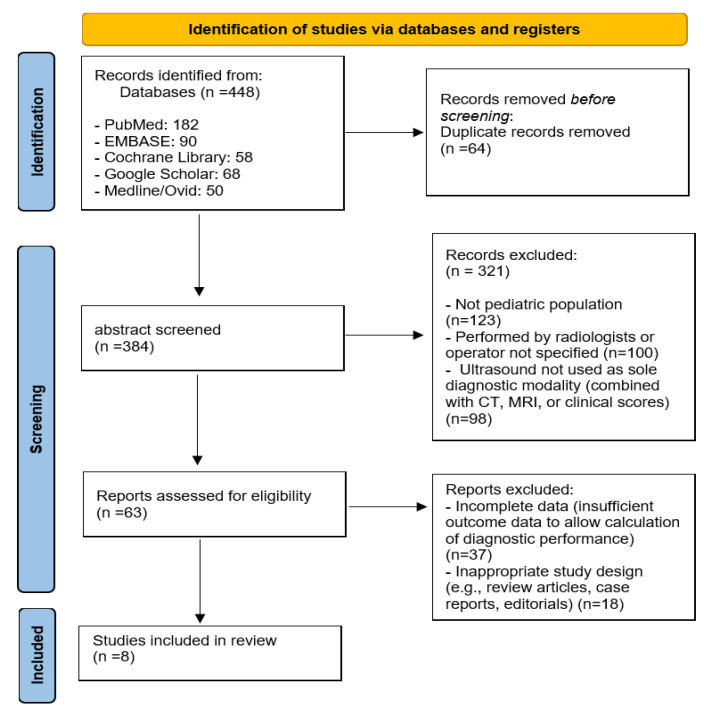
Diagram illustrating the study selection procedure.

A total of 448 articles were identified through the database search. After the initial screening, duplicates were removed, leaving 384 articles. The titles and abstracts of these 384 articles were reviewed, and 321 articles that did not meet the criteria were excluded. The full texts of the remaining 63 articles were thoroughly reviewed, and articles that did not provide diagnostic data such as sensitivity, specificity, positive predictive value (PPV), or negative predictive value (NPV) were excluded. Additionally, review articles, case reports, letters, comments, or studies where the ultrasound was performed by radiologists were excluded from consideration. The final analysis comprised eight studies that met the predefined inclusion criteria. (Figure 1).

### 2.5. Evaluation of Methodological Quality

We assessed the risk of bias and applicability concerns in the included studies using the Quality Assessment of Diagnostic Accuracy Studies-2 (QUADAS-2) tool [22]. This tool evaluates four domains: patient selection, index test, reference standard, and flow and timing. Each domain is rated for risk of bias and applicability concerns as “low,” “high,” or “unclear,” based on specific signaling questions. Detailed assessments are presented in Supplementary Table S1.

### 2.6. Synthesis and Analysis of Data

We extracted demographic characteristics and diagnostic performance data from each included study, including sensitivity, specificity, positive predictive value (PPV), and negative predictive value (NPV). A random-effects model was employed for all meta-analyses to account for potential clinical and methodological heterogeneity among studies. Although statistical heterogeneity was low in some cases, we considered it conceptually inappropriate to assume a common true effect size across all studies. The degree of heterogeneity was assessed using the I^2^ statistic. Forest plots and summary receiver operating characteristic (SROC) curves were generated to visually present the diagnostic performance of an ultrasound performed by non-radiologists in identifying acute appendicitis in pediatric patients. All statistical analyses were performed using Meta-Disc (version 1.4); ref. [23] Review Manager (RevMan, version 5.3); and the metaDTA online tool (https://crsu.shinyapps.io/metaDTA/) (accessed on 10 March 2025).

### 2.7. Methodological Quality Evaluation in the Included Studies

**Figure 2:**
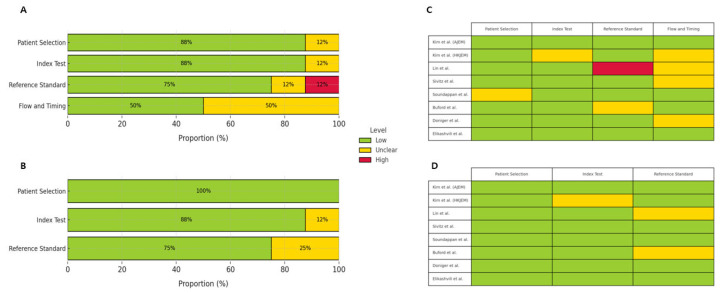
Overall quality assessment of included studies using the QUADAS-2 tool. (**A**) Risk of bias summary bar chart. (**B**) Applicability concerns summary bar chart. Each domain is presented as a percentage of included studies categorized as low risk (green), high risk (red), or unclear risk (yellow). (**C**) Traffic light plot for risk of bias across individual studies. (**D**) Traffic light plot for applicability concerns across individual studies. Each domain is color-coded according to the assessed risk level: low (green), unclear (yellow), or high (red) [8,10,11,12,13,14,15,16].

The information from the included studies, including the total number of enrolled patients, year of publication, sample size, journal name, first author, mean age of the patients, study design, ultrasound operator, and diagnostic outcomes (true positives, false positives, true negatives, and false negatives), is presented in Table 1 [8,10,11,12,13,14,15,16]. The methodological quality of the studies was assessed using the QUADAS-2 tool, with the results presented in Figure 2. Most studies showed a low risk of bias in the patient selection domain, while the index test and reference standard domains often demonstrated an unclear risk of bias. The two studies by Kim et al. included in this review were conducted at the same institution but involved different patient populations and study designs. The *American Journal of Emergency Medicine* (AJEM) study was a prospective observational study enrolling 115 pediatric patients, focusing on the diagnostic accuracy of clinician-performed and tele-mentored ultrasonography. In contrast, the *Hong Kong Journal of Emergency Medicine* (HKJEM) study was a retrospective study involving 166 pediatric patients, which assessed treatment decisions based on specific sonographic findings (periappendiceal fat infiltration and/or appendicolith) to reduce unnecessary surgeries [12,13].

## 3. Results

**Figure 3:**
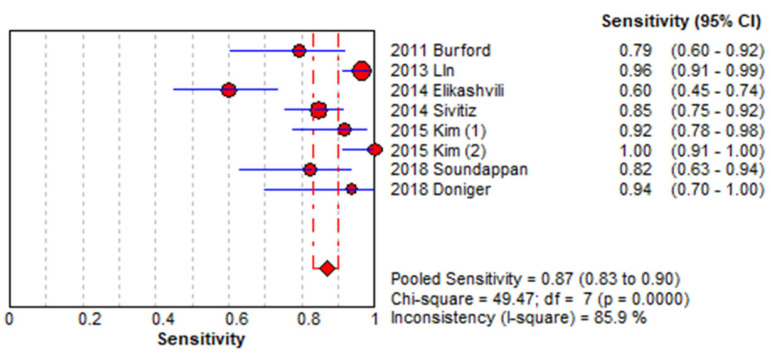
Forest plot showing sensitivity across included studies. Each study is identified by the first author’s name and year of publication. Horizontal lines represent 95% confidence intervals (CIs) [8,10,11,12,13,14,15,16].

**Figure 4:**
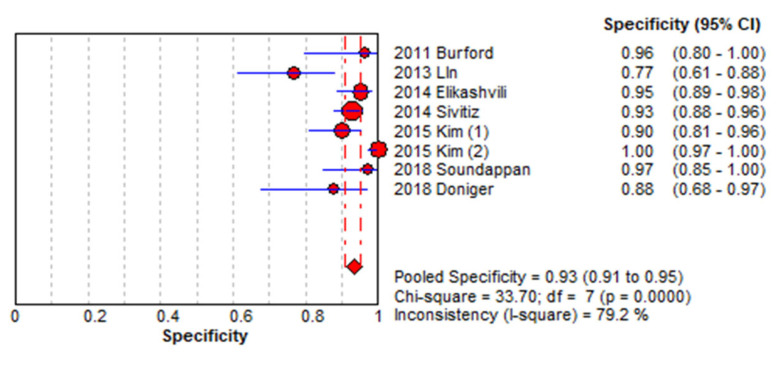
Forest plot showing specificity across included studies. Each study is identified by the first author’s name and year of publication. Horizontal lines represent 95% confidence intervals (CIs) [8,10,11,12,13,14,15,16].

**Figure 5:**
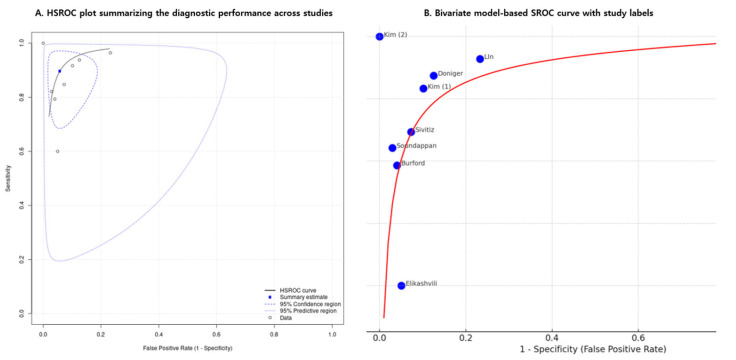
Summary receiver operating characteristic (SROC) curves for diagnostic accuracy. (**A**) Hierarchical summary receiver operating characteristic (HSROC) curve showing the summary point (blue square), 95% confidence region (dashed ellipse), and 95% prediction region (dotted ellipse). (**B**) SROC curve based on a bivariate random-effects model, with each study represented by a blue dot labeled with the study’s first author. The red line indicates the model-based summary curve, and the area under the curve (AUC) is 0.783 (95% CI: 0.708–0.853). AUC, area under the curve; CI, confidence interval [8,10,11,12,13,14,15,16].

Figure 3 and Figure 4 present forest plots displaying the sensitivity and specificity values along with their 95% confidence intervals for each included study. The overall pooled sensitivity was estimated at 0.87 (95% CI, 0.83–0.90), while the pooled specificity was 0.93 (95% CI, 0.91–0.95). Substantial heterogeneity was observed, with I^2^ values of 85.9% for sensitivity and 79.2% for specificity. Figure 5 presents the HSROC curve derived from a hierarchical summary model (Figure 5A), which includes the summary point (blue square), 95% confidence region (dashed blue ellipse), and 95% predictive region (dotted blue ellipse), with the SROC curve based on a bivariate random-effects model (Figure 5B), in which each blue dot represents an individual study labeled with the study’s first author and the red line indicates the model-based summary curve; both visualizations reflect between-study heterogeneity in sensitivity and specificity (Figure 5).

Using a bivariate random-effects meta-analysis model, the estimated area under the summary receiver operating characteristic (SROC) curve was 0.783, indicating moderate to good overall diagnostic accuracy. The 95% confidence interval for the AUC, calculated via nonparametric bootstrap resampling, ranged from 0.708 to 0.853. This model-based estimate accounts for between-study variability and provides a reliable measure of the test’s ability to distinguish between patients with and without acute appendicitis.

## 4. Discussion

Numerous studies have evaluated the diagnostic performance of ultrasound, and in adult populations, ultrasound examinations performed by non-radiologist physicians have demonstrated high accuracy [9,24,25,26,27]. Additionally, in pediatric patients, the diagnostic accuracy of ultrasound performed by professional sonographers or radiologists has been well-established [28,29].

However, despite this substantial body of evidence, limited research has assessed the diagnostic performance of ultrasound for pediatric appendicitis when performed by non-radiologist physicians. Previous systematic reviews and meta-analyses have generally reported high sensitivity and specificity for ultrasound in diagnosing acute appendicitis, but most studies have focused on adult populations and demonstrated substantial heterogeneity in findings [25,30,31]. Moreover, pediatric appendicitis often presents with more diverse and atypical symptoms compared to adults, making timely and accurate diagnosis particularly challenging [1].

In this systematic review and meta-analysis, ultrasound examinations conducted by non-radiologist physicians demonstrated moderate-to-good diagnostic performance in detecting acute appendicitis in pediatric patients. Prior research has suggested that the diagnostic accuracy of clinical examination alone for pediatric appendicitis is approximately 75%. In contrast, our meta-analysis demonstrated that non-radiologist-performed ultrasound yields a pooled sensitivity of 87% and specificity of 93%, reflecting a notable improvement in diagnostic performance. Given that clinical signs in children are often nonspecific or atypical, point-of-care ultrasound may serve as a valuable adjunct to enhance diagnostic confidence in the initial evaluation. In most included studies, ultrasound was selectively performed in children with suspected appendicitis based on initial clinical evaluation, rather than in all cases of abdominal pain. This highlights the continued importance of clinical examination as the foundation of the diagnostic process, with ultrasound serving as a valuable adjunct, particularly when clinical findings are ambiguous or inconclusive.

These findings are especially meaningful in emergency and resource-limited settings, where immediate access to radiologists may not always be feasible. Our study uniquely highlights that, with appropriate education and training, non-radiologist physicians can effectively utilize ultrasound to aid in the timely and accurate diagnosis of pediatric appendicitis. Notably, the relatively high negative predictive value (NPV) found in this analysis suggests that non-radiologist-performed ultrasound is effective in excluding acute appendicitis in children. This may contribute to minimizing unnecessary radiation exposure from additional imaging and reducing the number of avoidable surgical procedures.

However, it is important to note that the diagnostic performance of non-radiologist-performed ultrasound, with an AUC of 0.783 in our study, remains lower than that reported in previous meta-analyses of radiologist-performed imaging. For example, Eng et al. reported a pooled sensitivity and specificity of 91.3% and 95.2%, respectively, for radiologist-performed ultrasound in pediatric patients [32]. Furthermore, Binkovitz et al. conducted a large retrospective study evaluating the diagnostic performance of radiologist-performed ultrasound in 790 pediatric patients with suspected appendicitis. When a definitive interpretation was available, they reported a sensitivity of 94.8%, a specificity of 96.3%, and an overall diagnostic accuracy of 96% [28]. These findings further support the high accuracy of ultrasound examinations conducted and interpreted by radiologists in pediatric settings.

In contrast, while non-radiologist-performed ultrasound in our study demonstrated moderate-to-good diagnostic performance, the relatively lower sensitivity (87%) implies a higher risk of false-negative results. This is particularly concerning in pediatric appendicitis, where delayed diagnosis may lead to complications such as perforation. Therefore, while non-radiologist-performed ultrasound can serve as a practical diagnostic tool in resource-limited environments, clinicians should maintain a high index of suspicion and consider prompt surgical consultation or additional imaging when sonographic findings are inconclusive.

Nevertheless, the findings of this study suggest that an ultrasound performed by non-radiologist physicians can still achieve a clinically meaningful level of diagnostic accuracy.

This capability can significantly improve diagnostic efficiency in clinical environments where specialized imaging personnel are limited. These findings are especially important in healthcare settings where radiologists are not readily available, such as rural hospitals, community emergency departments, or low-resource countries. In such settings, delays in radiologist-performed imaging may increase the risk of complications due to diagnostic delays. Appropriately trained non-radiologist physicians can use ultrasound to improve diagnostic speed and patient care. For instance, emergency physicians with focused ultrasound training can help reduce unnecessary transfers and ensure timely diagnosis. Integrating point-of-care ultrasound into pediatric emergency care can further streamline clinical workflows in resource-limited environments.

These findings support the broader integration of point-of-care ultrasound (POCUS) into pediatric emergency care, particularly in institutions with limited access to on-site radiology services. To support such implementation, the American College of Emergency Physicians (ACEP) recommends a structured training pathway for POCUS credentialing that includes didactic instruction, hands-on scanning, and supervised image acquisition. A minimum of 25 to 50 supervised examinations per clinical application is advised to ensure competency [33]. The European Society of Paediatric Radiology (ESPR) likewise emphasizes the importance of standardized education, certification, and ongoing quality assurance to ensure the safe and effective use of POCUS by non-radiologist physicians. In South Korea, recent surveys of medical schools have identified limited training time and insufficient infrastructure as key barriers to incorporating POCUS education into undergraduate medical curricula [33,34,35].

Based on these standards, the clinical use of non-radiologist-performed ultrasound should be supported by institution-level training programs aligned with established guidelines. Regular quality assurance processes should also be implemented to maintain diagnostic accuracy and ensure patient safety. These measures are particularly important in emergency departments without full-time radiology coverage, where non-radiologist physicians may be required to perform time-sensitive ultrasound evaluations in pediatric patients.

Future initiatives should prioritize the development of standardized ultrasound training programs tailored to non-radiologist physicians, with the aim of improving diagnostic accuracy in pediatric emergency settings. Moreover, future research should evaluate the impact of non-radiologist-performed ultrasound on key clinical outcomes, such as diagnostic timeliness, length of hospital stay, and rates of unnecessary appendectomy. Recent advances in AI-assisted ultrasound interpretation and the development of compact, high-resolution devices provide new opportunities to enhance diagnostic performance, particularly when used by non-specialist providers. In this context, further studies may also explore the potential role of non-radiologist-performed POCUS in the differential diagnosis of pediatric abdominal conditions, including intussusception.

### Limitations

Our meta-analysis demonstrates that non-radiologist-performed ultrasound can be a useful tool in diagnosing acute appendicitis in pediatric patients. However, several limitations must be considered when interpreting these findings. One major limitation of this study is the heterogeneity among the included studies. There was considerable heterogeneity in sensitivity and specificity among the studies, suggesting that various factors, such as the experience and skill level of the ultrasound operators, as well as differences in equipment, may influence diagnostic accuracy for acute appendicitis in children. In pediatric cases, the accuracy of ultrasound examinations may be highly dependent on the operator’s level of expertise. Furthermore, the reporting of subject background, the exclusion criteria, and the intent of the screening process was unclear, and the number of included studies was small; several had limited sample sizes and did not implement blinding procedures, potentially affecting the overall reliability of the findings.

Additionally, some studies did not clearly explain the diagnostic methods used for evaluating non-visualized or ambiguous findings of the appendix, and descriptions of follow-up and additional testing were vague in certain cases. However, due to the small number of included studies, the uneven distribution of key variables such as operator type, study design, and geographic region, and the frequent lack of reporting on covariates such as the number of operators and types of ultrasound devices, it was not feasible to perform subgroup analyses or meta-regression. Accordingly, we included a qualitative discussion of potential sources of heterogeneity to support the interpretation of the study findings.

Another notable limitation is that this review was not prospectively registered in PROS PERO or any other systematic review registry, which may affect the transparency and reproducibility of the review process.

## 5. Conclusions

This review systematically analyzed studies that explored the diagnostic use of ultrasound performed by non-radiologist physicians for the diagnosis of acute appendicitis in children, and demonstrated that such ultrasound provides moderate-to-good sensitivity and specificity for diagnosing acute appendicitis. These findings support the use of non-radiologist-performed ultrasound as a valuable diagnostic tool for pediatric appendicitis, particularly in emergency settings and resource-limited environments with limited personnel.

However, given the substantial heterogeneity observed among the included studies, additional imaging or clinical follow-up may be necessary when ultrasound results are inconclusive. To reduce such heterogeneity in future research, it is important to standardize diagnostic criteria for appendicitis and ultrasound training protocols for operators, and to ensure consistent reporting of key covariates such as operator experience, patient characteristics, and imaging equipment. Multicenter prospective randomized controlled trials using unified methodologies would particularly help to clarify the diagnostic accuracy of ultrasounds performed by non-radiologist physicians and evaluate the impact of operator training levels more effectively.

## Figures and Tables

**Table 1:** Characteristics of the included studies.

Year	Author	Country	Sample Size	Age (Years)	Study Design	Operator	TP	FP	FN	TN	Sensitivity (%)	Specificity (%)	PPV(%)	NPV(%)
2018	Doniger [10]	United States	40	2–18 (range)	Prospective	EM physician	15	3	1	21	93.8	87.5	83.3	95.5
2018	Soundappan [11]	Australia	62	9.5 ± 2.8 (mean ± SD)	Prospective	Surgeon	23	1	5	33	82.1	97.0	95.8	86.9
2015	Kim (1) [12]	South Korea	115	Not reported	Prospective	EM physician or resident	33	8	3	71	91.7	89.9	80.5	95.6
2015	Kim (2) [13]	South Korea	166	10.6 (mean)	retrospective	EM physician	40	0	0	126	100	100	100	100
2014	Sivitz [8]	United States	264	10.2 (median); range 2–20.9	Prospective	EM physician	72	13	13	166	84.7	92.7	84.7	92.7
2014	Elikashvili [14]	United States	150	12.0 ± 5.2 (mean ± SD)	Prospective	EM physician	30	5	20	95	60.0	95.0	85.7	82.6
2013	Lin [15]	Taiwan	155	6 ± 5.8 (mean ± SD)	retrospective	EM physician	108	10	4	33	96.4	76.7	91.5	89.2
2011	Burford [16]	United States	54	8.8 (mean); range 3–16	Prospective	Surgeon	23	1	6	24	79.3	96.0	95.8	80.0

TP = true positive; FP = false positive; TN = true negative; FN = false negative; PPV = Positive Predictive Value; NPV = Negative Predictive Value SD = standard deviation; EM = emergency medicine.

## Data Availability

All data analyzed in this study were from previously published sources. No new data were generated.

## Supplementary Materials

The following supporting information can be downloaded at: https://www.mdpi.com/article/10.3390/medicina61071308/s1, Table S1: The QUADAS-2 Tool for the Quality Assessment of Diagnostic Accuracy Studies; Table S2: QUADAS-2 Assessment Summary for Included Studies.

## Abbreviations

The terms abbreviated in this paper are as follows:

CT	Computed Tomography
US	Ultrasound
PRISMA	Preferred Reporting Items for Systematic Reviews and Meta-Analyses
PubMed	US National Library of Medicine’s database of biomedical literature
Ovid MEDLINE	Online database of biomedical articles
EMBASE	Online database of biomedical articles
Cochrane Library	Collection of databases of systematic reviews and other evidence
QUADAS-2	Quality Assessment of Diagnostic Accuracy Studies—Second Edition
PPV	Positive Predictive Value
NPV	Negative Predictive Value
SROC	Summary Receiver Operating Characteristic curve
CI	Confidence Interval
POCUS	Point-of-care ultrasound

## References

[1] Bundy D.G., Byerley J.S., Liles E.A., Perrin E.M., Katznelson J., Rice H.E. (2007). Does this child have appendicitis?. JAMA.

[2] Addiss D.G., Shaffer N., Fowler B.S., Tauxe R.V. (1990). The epidemiology of appendicitis and appendectomy in the United States. Am. J. Epidemiol..

[3] Becker T., Kharbanda A., Bachur R. (2007). Atypical clinical features of pediatric appendicitis. Acad. Emerg. Med..

[4] Smith M.P., Katz D.S., Lalani T., Carucci L.R., Cash B.D., Kim D.H., Piorkowski R.J., Small W.C., Spottswood S.E., Tulchinsky M. (2015). ACR Appropriateness Criteria^®^ Right Lower Quadrant Pain—Suspected Appendicitis. Ultrasound Q..

[5] Aspelund G., Fingeret A., Gross E., Kessler D., Keung C., Thirumoorthi A., Oh Stephen P., Behr G., Chen S., Lampl B. (2014). Ultrasonography/MRI versus CT for diagnosing appendicitis. Pediatrics.

[6] Brenner D.J., Hall E.J. (2007). Computed tomography—An increasing source of radiation exposure. N. Engl. J. Med..

[7] Shahbazipar M., Seyedhosseini J., Vahidi E., Motahar Vahedi H.S., Jahanshir A. (2019). Accuracy of ultrasound exam performed by emergency medicine versus radiology residents in the diagnosis of acute appendicitis. Eur. J. Emerg. Med..

[8] Sivitz A.B., Cohen S.G., Tejani C. (2014). Evaluation of acute appendicitis by pediatric emergency physician sonography. Ann. Emerg. Med..

[9] Gungor F., Kilic T., Akyol K.C., Ayaz G., Cakir U.C., Akcimen M., Eken C. (2017). Diagnostic Value and Effect of Bedside Ultrasound in Acute Appendicitis in the Emergency Department. Acad. Emerg. Med..

[10] Doniger S.J., Kornblith A. (2018). Point-of-Care Ultrasound Integrated Into a Staged Diagnostic Algorithm for Pediatric Appendicitis. Pediatr. Emerg. Care.

[11] Soundappan S.S., Karpelowsky J., Lam A., Lam L., Cass D. (2018). Diagnostic accuracy of surgeon performed ultrasound (SPU) for appendicitis in children. J. Pediatr. Surg..

[12] Kim C., Kang B.S., Choi H.J., Lim T.H., Oh J., Chee Y. (2015). Clinical application of real-time tele-ultrasonography in diagnosing pediatric acute appendicitis in the ED. Am. J. Emerg. Med..

[13] Kim C., Kang B., Park J., Ha Y. (2015). The Use of Clinician-Performed Ultrasonography to Determine the Treatment Method for Suspected Paediatric Appendicitis. Hong Kong J. Emerg. Med..

[14] Elikashvili I., Tay E.T., Tsung J.W. (2014). The effect of point-of-care ultrasonography on emergency department length of stay and computed tomography utilization in children with suspected appendicitis. Acad. Emerg. Med..

[15] Lin W.C., Lin C.H. (2013). Re-appraising the role of sonography in pediatric acute abdominal pain. Iran J. Pediatr..

[16] Burford J.M., Dassinger M.S., Smith S.D. (2011). Surgeon-performed ultrasound as a diagnostic tool in appendicitis. J. Pediatr. Surg..

[17] Arredondo Montero J., Bardají Pascual C., Antona G., Ros Briones R., López-Andrés N., Martín-Calvo N. (2023). The BIDIAP index: A clinical, analytical and ultrasonographic score for the diagnosis of acute appendicitis in children. Pediatr. Surg. Int..

[18] Wu S., Gu F., Yu M., Zhu Z. (2025). Using a sum of the cross diameters of the appendix measured on ultrasonography as a criterion can more effectively predict acute appendicitis. Eur. Radiol..

[19] Xu Y., Jeffrey R.B., Shin L.K., DiMaio M.A., Olcott E.W. (2016). Color Doppler Imaging of the Appendix: Criteria to Improve Specificity for Appendicitis in the Borderline-Size Appendix. J. Ultrasound Med..

[20] Pogorelić Z., Mihanović J., Ninčević S., Lukšić B., Elezović Baloević S., Polašek O. (2021). Validity of Appendicitis Inflammatory Response Score in Distinguishing Perforated from Non-Perforated Appendicitis in Children. Children.

[21] Page M.J., McKenzie J.E., Bossuyt P.M., Boutron I., Hoffmann T.C., Mulrow C.D., Shamseer L., Tetzlaff J.M., Akl E.A., Brennan S.E. (2021). The PRISMA 2020 statement: An updated guideline for reporting systematic reviews. BMJ.

[22] Whiting P., Rutjes A.W., Reitsma J.B., Bossuyt P.M., Kleijnen J. (2003). The development of QUADAS: A tool for the quality assessment of studies of diagnostic accuracy included in systematic reviews. BMC Med. Res. Methodol..

[23] Zamora J., Abraira V., Muriel A., Khan K., Coomarasamy A. (2006). Meta-DiSc: A software for meta-analysis of test accuracy data. BMC Med. Res. Methodol..

[24] Lehmann B., Koeferli U., Sauter T.C., Exadaktylos A., Hautz W.E. (2022). Diagnostic accuracy of a pragmatic, ultrasound-based approach to adult patients with suspected acute appendicitis in the ED. Emerg. Med. J..

[25] Lee S.H., Yun S.J. (2019). Diagnostic performance of emergency physician-performed point-of-care ultrasonography for acute appendicitis: A meta-analysis. Am. J. Emerg. Med..

[26] Becker B.A., Kaminstein D., Secko M., Collin M., Kehrl T., Reardon L., Stahlman B.A. (2022). A prospective, multicenter evaluation of point-of-care ultrasound for appendicitis in the emergency department. Acad. Emerg. Med..

[27] Sharif S., Skitch S., Vlahaki D., Healey A. (2018). Point-of-care ultrasound to diagnose appendicitis in a Canadian emergency department. CJEM.

[28] Binkovitz L.A., Unsdorfer K.M., Thapa P., Kolbe A.B., Hull N.C., Zingula S.N., Thomas K.B., Homme J.L. (2015). Pediatric appendiceal ultrasound: Accuracy, determinacy and clinical outcomes. Pediatr. Radiol..

[29] Gilligan L.A., Trout A.T., Davenport M.S., Zhang B., O’Hara S.M., Dillman J.R. (2021). Predictors of Clinical Outcomes in Pediatric Appendicitis: Role of the Individual Sonographer and Radiologist When Using a First-Line Ultrasound Approach. J. Am. Coll. Radiol..

[30] Cho S.U., Oh S.K. (2023). Accuracy of ultrasound for the diagnosis of acute appendicitis in the emergency department: A systematic review. Medicine.

[31] Fields J.M., Davis J., Alsup C., Bates A., Au A., Adhikari S., Farrell I. (2017). Accuracy of Point-of-care Ultrasonography for Diagnosing Acute Appendicitis: A Systematic Review and Meta-analysis. Acad. Emerg. Med..

[32] Eng K.A., Abadeh A., Ligocki C., Lee Y.K., Moineddin R., Adams-Webber T., Schuh S., Doria A.S. (2018). Acute Appendicitis: A Meta-Analysis of the Diagnostic Accuracy of US, CT, and MRI as Second-Line Imaging Tests after an Initial US. Radiology.

[33] Tayal V.S., Raio C., Mandavia D. (2017). Ultrasound Guidelines: Emergency, Point-of-Care and Clinical Ultrasound Guidelines in Medicine. Ann. Emerg. Med..

[34] Whitehead A., Fullerton K., Miller H.C. (2021). Minimizing Ionizing Radiation in Evaluating Suspected Appendicitis in Children Before and After the Release of the ACEP Clinical Policy. Pediatr. Emerg. Care.

[35] Yoo J., Kang S.Y., Jo I.J., Kim T., Lee G.T., Park J.E., Lee S.U., Hwang S.Y., Cha W.C., Shin T.G. (2024). Status and perception of point-of-care ultrasound education in Korean medical schools: A national cross-sectional study. Medicine.

