# Supplementary material for: Diagnostic Accuracy of Non-Radiologist-Performed Ultrasound for Diagnosing Acute Appendicitis in Pediatric Patients: A Systematic Review and Meta-Analysis
Source: Medicina (Kaunas). 2025 Jul 21;61(7):1308. doi: 10.3390/medicina61071308 (PMC12299215; doi:10.3390/medicina61071308)
Supplement: Supplementary file 1 [file medicina-61-01308-s001.zip › Detailed Search Strategies for Each Database.pdf]

Database	Search Terms Used	Filters Applied
**PubMed**	("ultrasound"[MeSH Terms] OR "ultrasonography" OR "sonography" OR "US" OR "USG") AND ("appendicitis"[MeSH Terms] OR "appendix") AND ("child"[MeSH Terms] OR "children" OR "pediatric")	English; Human; Up to June 2024
**Ovid MEDLINE**	(ultrasound OR ultrasonography OR sonography OR US OR USG).mp. AND (appendicitis OR appendix).mp. AND (child OR children OR pediatric).mp.	English; Human; Up to June 2024
**EMBASE**	('ultrasound'/exp OR 'ultrasonography' OR 'sonography' OR 'US' OR 'USG') AND ('appendicitis'/exp OR 'appendix') AND ('child'/exp OR 'children' OR 'pediatric')	English; Human; Up to June 2024
**Cochrane Library**	(ultrasound OR ultrasonography OR sonography OR US OR USG) in Title Abstract Keyword AND (appendicitis OR appendix) in Title Abstract Keyword AND (child OR children OR pediatric) in Title Abstract Keyword	No date restriction; English
**Google Scholar**	allintitle: ultrasound OR ultrasonography OR sonography OR US OR USG AND appendicitis OR appendix AND child OR children OR pediatric	First 100 results screened manually
