# Supplementary material for: Diagnostic Accuracy of Non-Radiologist-Performed Ultrasound for Diagnosing Acute Appendicitis in Pediatric Patients: A Systematic Review and Meta-Analysis
Source: Medicina (Kaunas). 2025 Jul 21;61(7):1308. doi: 10.3390/medicina61071308 (PMC12299215; doi:10.3390/medicina61071308)
Supplement: Supplementary file 1 [file medicina-61-01308-s001.zip › supplement table 1.pdf]

Table S1 The QUADAS-2 Tool for the Quality Assessment of Diagnostic Accuracy Studies

Item		Yes	No	Unclear
1.	Was the spectrum of patients representative of the patients who will receive the test in practice?			
2.	Were selection criteria clearly described?			
3.	Is the reference standard likely to correctly classify the target condition?			
4.	Is the time period between reference standard and index test short enough to be reasonably sure that the target condition did not change between the two tests?			
5.	Did the whole sample or a random selection of the sample, receive verification using a reference standard of diagnosis?			
6.	Did patients receive the same reference standard regardless of the index test result?			
7.	Was the reference standard independent of the index test (i.e. the index test did not form part of the reference standard)?			
8.	Was the execution of the index test described in sufficient detail to permit replication of the test?			
9.	Was the execution of the reference standard described in sufficient detail to permit its replication?			
10.	Were the index test results interpreted without knowledge of the results of the reference standard?			
11.	Were the reference standard results interpreted without knowledge of the results of the index test?			
12.	Were the same clinical data available when test results were interpreted as would be available when the test is used in practice?			
13.	Were uninterpretable/ intermediate test results reported?			
14.	Were withdrawals from the study explained?			

QUADAS-II= Quality Assessment of Diagnostic Accuracy Studies - Second Edition
