# Supplementary material for: Diagnostic Accuracy of Non-Radiologist-Performed Ultrasound for Diagnosing Acute Appendicitis in Pediatric Patients: A Systematic Review and Meta-Analysis
Source: Medicina (Kaunas). 2025 Jul 21;61(7):1308. doi: 10.3390/medicina61071308 (PMC12299215; doi:10.3390/medicina61071308)
Supplement: Supplementary file 1 [file medicina-61-01308-s001.zip › supplement table 2.pdf]

Supplementary Table S2. QUADAS-2 Assessment Summary for Included Studies

Summary of risk of bias and applicability concerns according to the QUADAS-2 tool across the eight included studies.

Study (Author, Year)	Patient Selection (Risk of Bias)	Index Test (Risk of Bias)	Reference Standard (Risk of Bias)	Flow and Timing (Risk of Bias)	Applicability: Patient Selection	Applicability: Index Test	Applicability: Reference Standard
Burford et al., 2011	Low	Low	Unclear	Low	Low	Low	Low
Doniger et al., 2016	Low	Low	Low	Unclear	Low	Low	Low
Elikashvili et al., 2014	Low	Low	Low	Low	Low	Low	Low
Kim et al., 2015 (1)	Low	Low	Low	Low	Low	Low	Low
Kim et al., 2015 (2)	Low	Unclear	Low	Unclear	Low	Unclear	Low
Lin et al., 2013	Low	Low	High	Unclear	Low	Low	Unclear
Sivitz et al., 2014	Low	Low	Low	Unclear	Low	Low	Low
Soundappan et al., 2018	Unclear	Low	Low	Low	Low	Low	Low

Note: Each study was assessed using the QUADAS-2 tool across four domains of bias (Patient Selection, Index Test, Reference Standard, and Flow and Timing) and three domains of applicability. Risk of bias and applicability concerns were rated as “Low,” “High,” or “Unclear.” Assessments were performed independently by the reviewing author.
